# Seryl tRNA synthetase cooperates with POT1 to regulate telomere length and cellular senescence

**DOI:** 10.1038/s41392-019-0078-1

**Published:** 2019-11-29

**Authors:** Yingxi Li, Xiyang Li, Mei Cao, Yuke Jiang, Jie Yan, Ze Liu, Rongcun Yang, Xu Chen, Peiqing Sun, Rong Xiang, Longlong Wang, Yi Shi

**Affiliations:** 1 School of Medicine, Nankai University, 94 Weijin Road, Tianjin, 300071 China; 2 Tianjin Key Laboratory Human Development and Reproductive Regulation, Nankai University Affiliated Hospital of Obstetrics and Gynecology, Tianjin, China; 3 Department of Cancer Biology, Wake Forest Comprehensive Cancer Center, Wake Forest School of Medicine, Winston-Salem, NC USA

**Keywords:** Senescence, Cancer genomics

## Abstract

Deregulated telomere length is a causative factor in many physiological and pathological processes, including aging and cancer. Many studies focusing on telomeres have revealed important roles for cooperation between the Shelterin protein complex and telomerase in maintaining telomere length. However, it remains largely unknown whether and how aging-related stresses, such as deregulated protein homeostasis, impact telomere length. Here, we explored the possible roles of aminoacyl tRNA synthetases (AARSs), key enzymes catalyzing the first reactions in protein synthesis, in regulating telomere length and aging. We selected seryl tRNA synthetase (SerRS) since our previous studies discovered expanded functions of SerRS in the nucleus in addition to its canonical cytoplasmic role in protein synthesis. In this study, we revealed that overexpression of SerRS promoted cellular senescence and inhibited the growth of cervical tumor xenografts in mice by triggering the senescence of tumor cells. In the nucleus, SerRS directly bound to telomeric DNA repeats and tethered more POT1 proteins to telomeres through a direct interaction between the UNE-S domain of SerRS and the OB1 domain of POT1. We further demonstrated that SerRS-induced enrichment of POT1 prevented the recruitment of telomerase to telomeres, resulting in progressive telomere shortening. Our data suggested a possible molecular link between protein synthesis and telomere length control, the deregulation of which may be associated with aging and cancer.

## Introduction

Human telomeres comprise tandem repeats of double-stranded TTAGGG repeats that terminate in a single-stranded 3' overhang. In human somatic cells, telomere sequence is progressively lost with each cell division, and this shortening may eventually trigger cell senescence.^1,2^ Deregulation of telomere-length maintenance has been observed in cancer and aging.^3^ In stem cells and cancer cells, which are able to maintain telomere length during cell division, a specialized reverse transcriptase named telomerase adds telomeric repeats to the chromosome ends.^4^ Overexpression of telomerase, which is usually not expressed in normal cells, results in an increased incidence of spontaneous cancer in mice.^5,6^ In addition to telomerase, the length of telomeres is also controlled by a telomere complex composed of six proteins, known as Shelterin, which binds to and protects the telomere from activating DNA damage signaling and double-strand break repair pathways.^7^

As a key protein complex regulating the recruitment of telomerase to telomeres, Shelterin is composed of six proteins, namely, TRF1, TRF2, TIN2, RAP1, POT1, and TPP1.^8–10^ In the Shelterin complex, POT1 is the only protein that binds single-stranded telomeric DNA with high affinity and specificity.^11,12^ This binding is mediated by the two oligonucleotide/oligosaccharide binding folds (OB-folds) of POT1 at the N-terminus, while the C-terminal portion of POT1 binds TPP1.^13^ Recent studies have indicated that both POT1 and TPP1 are critical factors for the recruitment of telomerase. POT1 has been shown to negatively regulate telomerase engagement. Depletion of POT1 or overexpression of OB1-truncated POT1 causes rapid telomerase-dependent telomere elongation.^14^ TPP1 can directly interact with telomerase through a protein surface termed the TEL patch.^15^ Cells lacking the TPP1/telomerase interaction undergo progressive telomere shortening.^16,17^ However, it remains largely unknown whether there are other regulators that bridge aging-related intrinsic and extrinsic stimuli to control telomere length.

Protein synthesis has been shown to affect lifespan. Many mutations in or depletions of the translational machinery were found to impact the lifespan of various organisms.^18–21^ However, how altered translation machinery affects lifespan is not fully understood. The nutrient-responsive target of rapamycin (TOR) signaling pathway, which has a conserved role in modulating longevity, has distinct roles in regulating translation initiation and elongation.^22–25^ As a key family of enzymes that catalyze the first reaction in protein synthesis, namely, adding amino acids to specific tRNAs, aminoacyl tRNA synthetases (AARSs) gained many new domains during evolution, expanding their functions beyond translation.^26^ Interestingly, some members, such as leucyl tRNA synthetase (LeuRS), have been reported to mediate the amino acid signals to the TOR pathway.^27^ We were specifically interested in another member, i.e., seryl tRNA synthetase (SerRS). Our previous studies have shown that vertebrate SerRS acquired a C-terminal domain unique to SerRS (namely, the UNE-S domain), which harbors a nuclear localization signal (NLS) and may divert a portion of SerRS proteins into the nucleus.^28^ Nuclear SerRS can directly bind the promoter of *VEGFA*, suppressing its transcription and therefore inhibiting angiogenesis.^29–31^ Our unpublished chromatin immunoprecipitation (ChIP) data suggested that SerRS may bind more DNA loci on regions of chromosomes, including telomeres. Here, we explored the impact of SerRS on cellular senescence and investigated the underlying SerRS-mediated regulation of telomere length through its interaction with telomeric DNA and Shelterin proteins.

## Results

### Nuclear SerRS decreases the length of telomeres and promotes cellular senescence

**Fig. 1:**
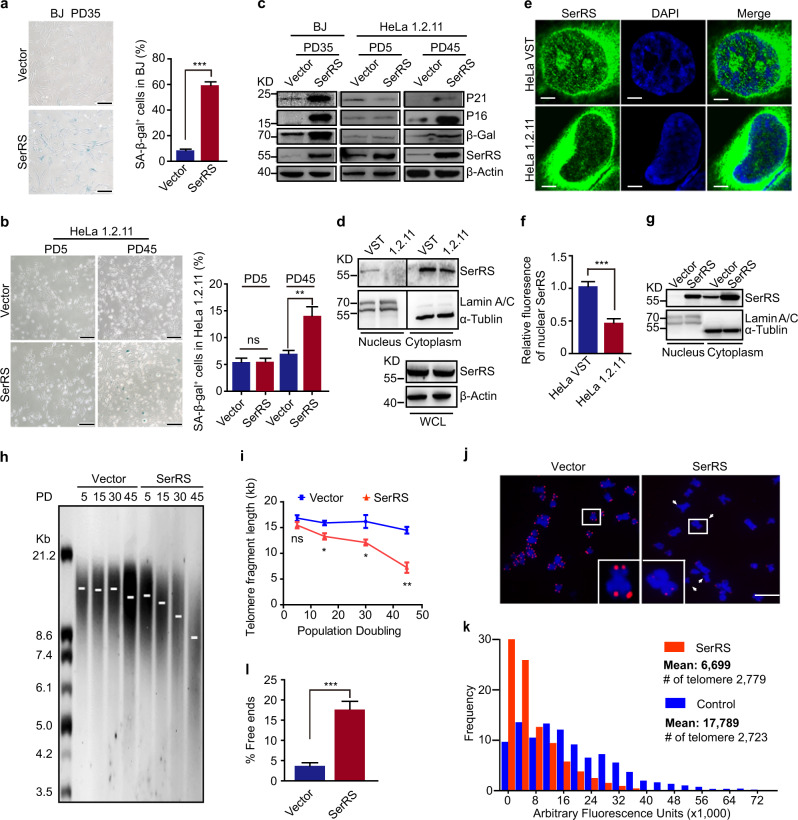
Nuclear SerRS promotes cellular senescence and telomere shortening. **a** SA-β-gal activity determined by X-gal staining in 35 population doublings (PD35) of BJ cells stably transfected with SerRS (SerRS) or empty vector (Vector). Scale bars represent 100 μm. Quantification of the percentage of SA-β-gal positive cells (SA-β-gal^+^ cells) is shown in the right panel. Data are represented as the means ± SEM (*n* = 10). ***P < 0.001, two-tailed Student’s *t*-test. **b** SA-β-gal activity assay on different PDs of HeLa 1.2.11 cells stably transfected with SerRS or empty vector. Quantification of the percentages of SA-β-gal^+^ cells is shown in the right panel (means ± SEM, *n* = 10, **P < 0.01, two-tailed Student’s *t*-test). **c** Immunoblot showing the levels of markers of cellular senescence in BJ or HeLa 1.2.11 stable cells at the indicated PDs. **d** The protein levels of SerRS in the cytoplasmic and nuclear fractions extracted from HeLa cells with very short telomeres (VST) or long telomeres (1.2.11) were analyzed by immunoblot. Lamin A/C and α-tubulin served as nuclear and cytoplasmic markers, respectively. WCL, whole cell lysate. **e** Nuclear SerRS proteins in HeLa VST and HeLa 1.2.11 are shown by immunofluorescent staining. Scale bars represent 5 μm. **f** Quantification of the relative levels of nuclear SerRS protein shown in (**e**). Data are represented as the means ± SEM (*n* = 10). ***P < 0.001, two-tailed Student’s t-test. **g** Immunoblot to show the levels of SerRS proteins in the cytoplasmic and nuclear fractions of indicated HeLa 1.2.11 stable cells. **h** HeLa 1.2.11 cells stably transfected with the SerRS expression vector or the empty vector were continuously passaged for the indicated PDs. The telomere lengths were examined by telomere restriction fragment (TRF) analysis. **i** The average telomere lengths from **h** were quantified by GelPro analysis software. Data are represented as the means ± SEM (*n* = 3). *P < 0.05, **P < 0.01, two-tailed Student’s *t*-test. **j** Representative images of the telomeres from metaphase chromosomes in indicated HeLa 1.2.11 cells analyzed by Q-FISH assay using a telomeric Cy3-(CCCTAA)_3_ PNA probe (red). Signal-free ends are indicated by arrows. Insets are magnified examples of individual chromosome. **k** Q-FISH images in **j** were quantified by TFL-TELO software. The distributions of individual telomeres with different lengths, which are represented by arbitrary fluorescence units, from a total of 20 metaphase cells per treatment were displayed. **l** Quantification of chromosome ends lacking a detectable telomeric signal in **j**, which is indicative of telomere shortening. Data are shown as the means±SEM (*n* = 15) from three independent experiments. ***P < 0.001, two-tailed Student’s *t*-test

It has been reported that the rate of protein synthesis decreases during cellular aging due to the declined efficiency of the components in the protein synthetic machinery, including ribosomes and elongation factors.^18^ However, as the key enzymes catalyzing the first reaction in protein synthesis, i.e., adding amino acids to their cognate tRNAs, the role of AARSs in aging remains largely unknown. To obtain insights into this question, we studied one AARS, SerRS, to test whether AARSs regulates cellular senescence. We overexpressed SerRS in normal fibroblasts, BJ cells, and in a HeLa cell strain, HeLa 1.2.11, which harbors long telomere repeats (~20 kb). We observed an unexpected increase in SA-β-gal activity (Fig. 1a, b) and increased levels of cellular senescence signaling molecules, such as P21, P16, and β-galactosidase (β-Gal)^32–34^ at late cell passages of both cells (Fig. 1c); these changes were even observed in HeLa 1.2.11 cells, which undergo little replicative senescence (Fig. 1b), suggesting a role of SerRS in promoting cellular senescence beyond its role in translation. Our previous studies have shown that in addition to its canonical function in protein biosynthesis, vertebrate SerRS evolved a noncanonical nuclear function to directly bind DNA and regulate the transcription of target genes involved in angiogenesis.^28^ We hypothesized that nuclear SerRS might promote cellular senescence. Given that telomere length control is a key event that triggers cellular senescence, we first examined the correlation between nuclear SerRS and telomere length.

We chose two strains of telomerase-positive HeLa cells that differ in telomere length, i.e., HeLa VST (for Very Short Telomeres) cells with an average telomere length of ~5 kb and HeLa 1.2.11 cells, which have longer telomeres (~20 kb). We observed similar levels of cytoplasmic SerRS proteins in both cells (Fig. 1d). However, as reported,^28^ we observed ~10% SerRS proteins in the nucleus of HeLa VST cells, while there were much lower levels of nuclear SerRS proteins in HeLa 1.2.11 cells (Fig. 1d). These results were further confirmed by immunofluorescent staining and confocal microscopy (Fig. 1e, f), suggesting that the different lengths of telomeres in these two cell strains may relate to the difference in levels of nuclear SerRS.

To further investigate whether nuclear SerRS regulated the length of telomeres, we established a stable SerRS-overexpressing HeLa 1.2.11 cell strain that showed increased SerRS expression in both the nucleus and the cytoplasm (Fig. 1g). We continuously passaged the cells for 45 population doublings (PD). Strikingly, the telomere length detected by Telomere Restriction Fragment (TRF) analysis showed a progressive reduction (from ~16 kb to ~9 kb) in SerRS-overexpressing HeLa 1.2.11 cells when compared with empty vector transfected cells, which showed almost no change in the length of telomere (Fig. 1h, i). The telomere length was also examined by the metaphase chromosome spreads followed by a telomeric quantitative fluorescent *in situ* hybridization (Q-FISH) assay (Fig. 1j, k). Significant telomere shortening was viewed by reduced FISH signals, further indicating that telomeres were globally shortened when SerRS was overexpressed. Consistently, we also observed a significant increase in the appearance of telomere-free chromosome ends, which is also indicative of telomere shortening, in SerRS-overexpressing cells (Fig. 1l).

Taken together, these results suggest that nuclear SerRS negatively regulated telomere length and thus led to cellular senescence.

### SerRS induces tumor cell senescence to inhibit the growth of cervical cancer xenografts in mice, and its expression correlates with better prognosis in cancer patients

**Fig. 2:**
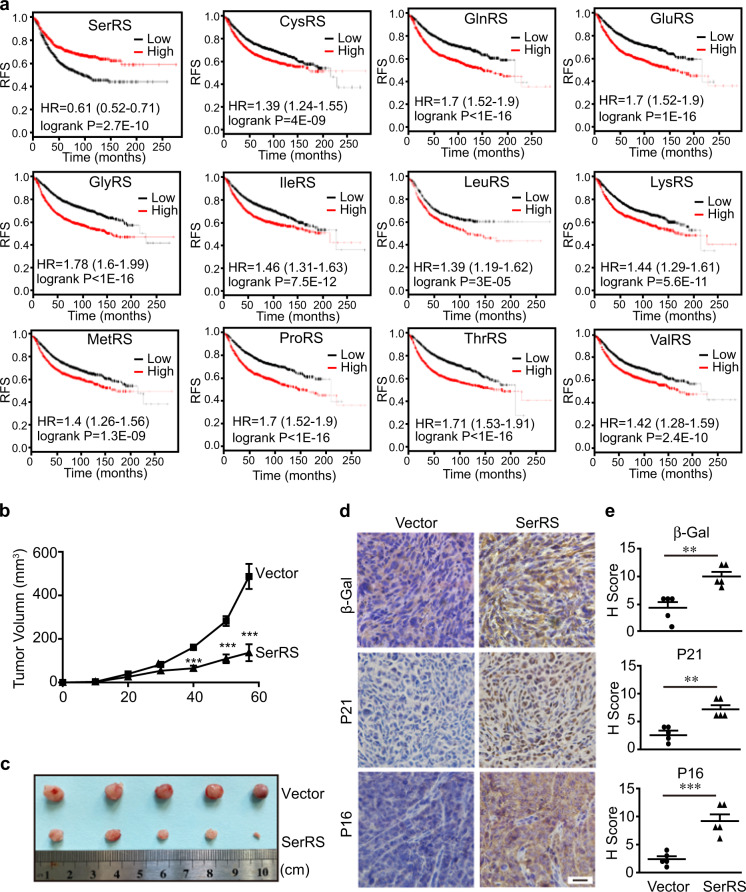
SerRS functions as a tumor suppressor and correlates with better prognosis of cancer patients. **a** Kaplan–Meier plots and hazard ratio analysis of human tRNA synthetases reveal a tight correlation with relapse-free survival (RFS) of breast cancer patients. Patient samples were divided into two halves as “low-expression” (black)” and “high-expression” (red) sets for each tRNA synthetase in the analysis (n = 1764). **b**, **c** HeLa cells stably transfected with the SerRS expression vector or the empty vector were injected subcutaneously into NOD/SCID mice (*n* = 5 in each group), and the growth curves of the tumor xenograft were monitored **b** (***P <0.001, two-tailed Student’s *t*-test). The dissected tumor xenografts are shown in **c**. **d**, **e** Immunohistochemistry analysis of β-Gal, P21 and P16 to show cellular senescence in the indicated xenografts **d**, and the protein levels were quantified based on the H-score **e** (**p < 0.01, ***p < 0.001)

Tumors require the active biosynthesis of macromolecules, including proteins, to fuel tumor cell growth and proliferation. We analyzed the correlation between the levels of AARSs and the relapse-free survival (RFS) of breast cancer patients in previously generated microarray data sets from 1764 breast cancer patients.^35^ As expected, high expression of many AARS members tightly correlated with a poor prognosis of cancer patients (Fig. 2a). Other AARS family members, except SerRS, showed no such tight correlation (Supplementary Fig. 1). In contrast, high expression of SerRS shows a very tight correlation with better prognosis of cancer patients (Fig. 2a), suggesting a novel role of SerRS in addition to protein biosynthesis in suppressing tumor progression. Consistently, we observed that the overexpression of SerRS induced the senescence of HeLa cells (Fig. 1b, c). These results further supported an important function of SerRS in balancing protein synthesis and telomere shortening-induced cellular senescence to prevent the malignant proliferation of cells.

To further test whether SerRS could suppress tumor progression in vivo, we used a tumor xenograft system; HeLa cells that were stably transfected with SerRS or an empty vector were xenografted into immune-deficient mice and monitored. Overexpression of SerRS in HeLa cells dramatically inhibited the growth of the tumor xenograft (Fig. 2b, c). The levels of the senescence molecular markers β-Gal, P21, and P16 were significantly increased in SerRS-overexpressing tumor cells in the tumor xenografts (Fig. 2d, e), suggesting that a high level of SerRS was able to induce the senescence of tumor cells, which led to the inhibited growth of tumor xenografts in mice. Taken together, the in vivo data further confirmed that SerRS may function as a tumor suppressor by promoting cellular senescence.

### SerRS directly binds telomeric repeats

**Fig. 3:**
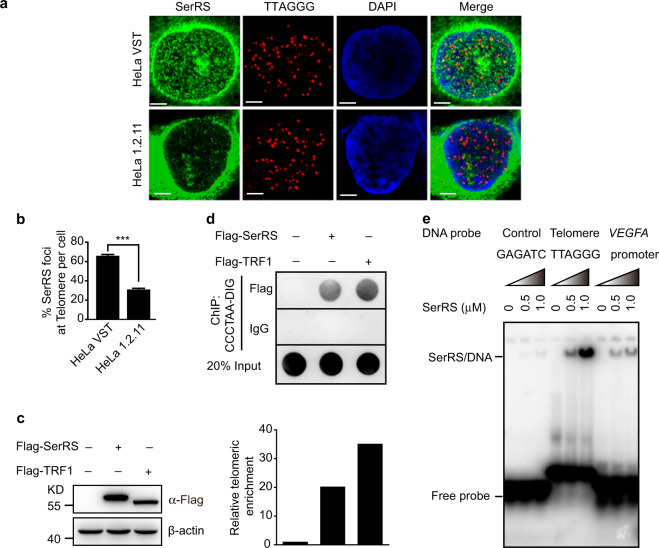
SerRS binds telomeres by direct interaction with telomeric DNA. **a** SerRS (green) and telomeres (red) in HeLa VST cells were visualized by immunofluorescent staining and FISH, respectively. Scale bars represent 5 μm. **b** Quantification of the data shown in **a** to show the percentage of SerRS-associated telomeric foci per cell. Data are represented as the means ± SEM (*n* = 20). ***P < 0.001, two-tailed Student’s *t*-test. **c**, **d** Flag-tagged SerRS (Flag-SerRS) or Flag-tagged TRF1 (positive control) was ectopically expressed in HeLa 1.2.11 cells **c**. SerRS and TRF1-associated telomeric DNA fragments were immunoprecipitated with anti-Flag antibody or control IgG and analyzed by dot-blots with DIG-labeled (CCCTAA)_6_ probes (**d**, upper panel). The dot signals were quantified (**d**, lower panel). **e** EMSA to determine the direct binding between SerRS and telomeric DNA

Next, we explored how SerRS regulated the length of telomeres. We first investigated whether nuclear SerRS was able to bind to telomeres directly. The colocalization of SerRS with telomeres was examined by immunofluorescent staining for SerRS and FISH for telomeres in HeLa cells. We found that in the nucleus of HeLa VST cells, in addition to binding the promoters of target genes such as *VEGFA*,^29^ there was a portion of SerRS protein that localized to telomeres, whereas in HeLa 1.2.11 cells, there was much less SerRS protein in the nucleus (Fig. 3a, b). To further confirm the binding of SerRS to telomeres, Flag-tagged SerRS or Flag-tagged Shelterin TRF1 (as a positive control) was transfected into HeLa 1.2.11 cells (Fig. 3c). ChIP assays were performed with nuclear extracts using an anti-Flag antibody or a negative control IgG. The precipitated DNA fragments were then hybridized with a digoxin-labeled telomere-specific probe. As shown in Fig. 3d, the SerRS antibody coprecipitated telomere DNA fragments, as did the TRF1 antibody, suggesting the binding of SerRS to telomeres.

Given the capacity of SerRS to directly interact with DNA,^29^ we next examined whether SerRS directly interacted with telomeric DNA repeats. Using an electrophoretic mobility shift assay (EMSA) with purified recombinant SerRS protein, we found that SerRS specifically bound telomeric repeats and its known DNA-binding sequence from the *VEGFA* promoter,^29^ whereas there was no binding of SerRS with a random DNA sequence (Fig. 3e). Thus, SerRS could bind the telomere through direct interaction with telomeric DNA repeats.

### SerRS directly interacts with Shelterin POT1

The Shelterin complex has been shown to cooperate with telomerase to maintain telomere length homeostasis.^36^ Among the six proteins in Shelterin, POT1 was reported to bind a single-stranded telomere 3′ overhang via its N-terminal OB fold and therefore inhibit the recruitment of telomerase. Depletion of POT1 leads to rapid elongation of telomeres in telomerase-positive cells.^37^ In a high-throughput protein-protein interaction screening for Shelterin-associated proteins, SerRS was identified as a candidate protein that may interact with POT1.^38^

**Fig. 4:**
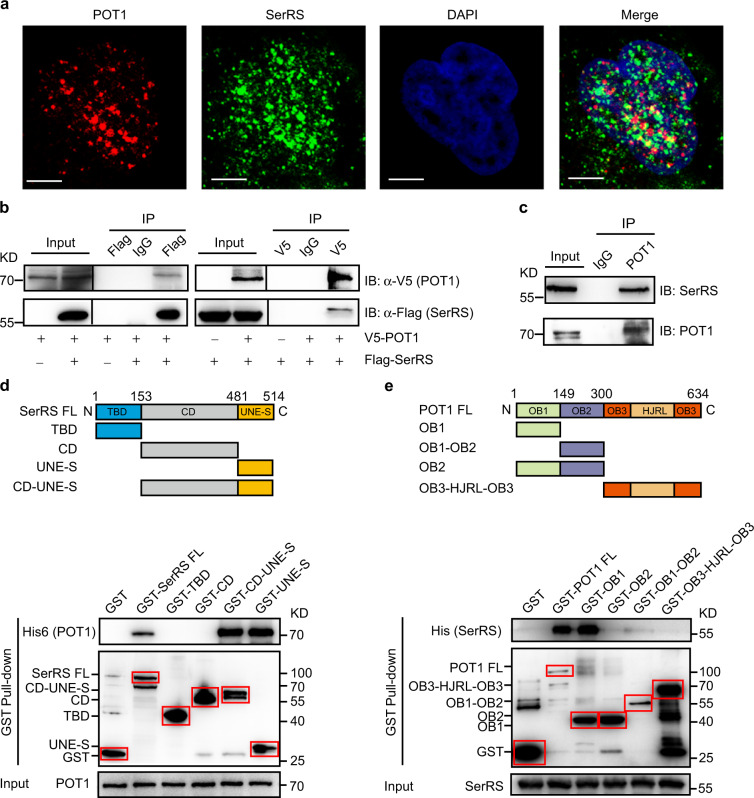
SerRS directly interacts with Shelterin POT1. **a** Immunofluorescent staining to show the colocalization of SerRS (green) and POT1 (red) in the nucleus of HeLa VST cells. Scale bars represent 5 μm. **b** Flag-tagged SerRS (Flag-SerRS) and V5-tagged POT1 (V5-POT1) vectors were transfected into HeLa cells, and immunoprecipitation (IP) was performed with the indicated antibodies. Cell lysates and immunoprecipitated proteins were analyzed by immunoblotting (IB) with the indicated antibodies. **c** HeLa VST cell lysates were immunoprecipitated with a POT1 antibody or a nonspecific IgG and analyzed by immunoblotting with the indicated antibodies. **d** A GST tag was fused with full length (FL) SerRS or the tRNA-binding domain (TBD), catalytic domain (CD) and unique to SerRS domain (UNE-S) of SerRS (upper panel). The GST-fused SerRS proteins were used to pull down purified His6-tagged POT1, and they were analyzed by immunoblot with anti-His6 antibody and anti-GST antibody (lower panel). **e** GST pull-down assays showed the direct interaction between SerRS and different domains of POT1. Full-length (FL) POT1 and its domains were fused with GST at the N-terminus to pull down purified His6-tagged SerRS. OB1, OB2 and OB3 stand for oligonucleotide/oligosaccharide DNA-binding domains 1, 2, and 3; HJR stands for Holliday junction resolvase-like domains

To confirm the interaction between SerRS and POT1, we first examined the localization of these two proteins in HeLa VST cells. SerRS was partially colocalized with POT1 in the nucleus (Fig. 4a). The interaction between SerRS and POT1 was further confirmed by their Co-IP from HeLa cells. As shown in Fig. 4b, V5-tagged POT1 was able to be coprecipitated with Flag-tagged SerRS via Flag antibody-mediated isolation; the reverse experiment with a V5 antibody produced a complementary result. In HeLa VST cells, the endogenous SerRS proteins could be coprecipitated with endogenous POT1 by POT1 antibody (Fig. 4c). Taken together, these results strongly suggested that SerRS interacted with POT1 in the nucleus.

To examine whether SerRS directly interacts with POT1, we purified recombinant POT1 with a His6 tag at its C-terminal end and recombinant GST-fused SerRS proteins. The GST pull-down assay clearly showed the direct interaction between SerRS and POT1 (Fig. 4d). Further domain mapping showed that SerRS interacts with POT1 through the UNE-S domain (Fig. 4d), which harbors an NLS that was appended to vertebrate SerRS during evolution.^28^ Our domain mapping assay also showed that POT1 bound to SerRS through its OB1 domain (Fig. 4e), through which POT1 bound to single-stranded telomere DNA.^39^

Taken together, these results suggest that SerRS directly interacts with POT1 in the nucleus and may regulate telomere length through POT1.

### SerRS tethers more POT1 to telomeres

**Fig. 5:**
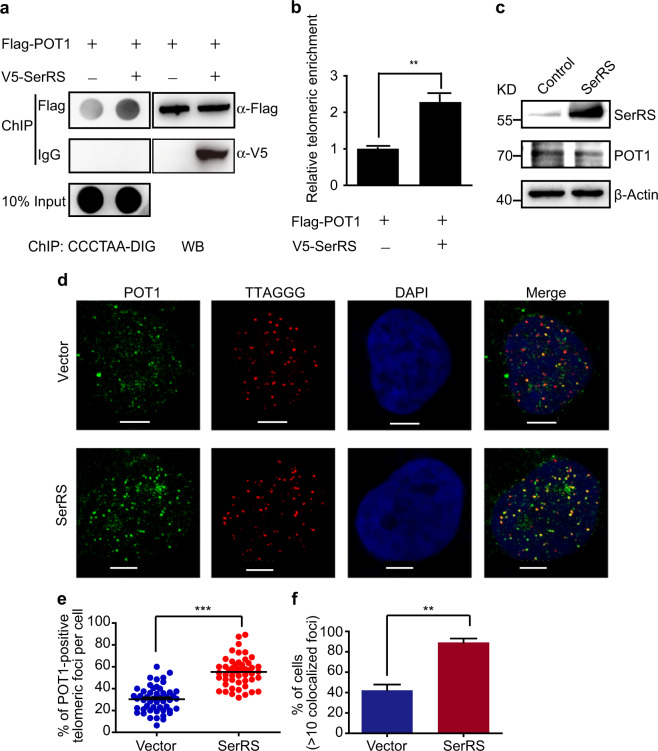
SerRS promotes the recruitment of POT1 to telomeres. **a** Flag-tagged POT1 was transfected into HeLa 1.2.11 cells with V5-tagged SerRS or empty vectors (right panel). POT1-associated telomeric DNA fragments were immunoprecipitated with an anti-Flag antibody or a control IgG and analyzed by dot blot using DIG-labeled (CCCTAA)_6_ probes (left panel). **b** Quantification of the data shown in **a**. Data are represented as the means ± SEM (*n* = 3). **P < 0.005, two-tailed Student’s *t*-test. **c** The levels of POT1 expression in SerRS-overexpressing HeLa 1.2.11 cells as determined by immunoblot analysis. **d** IF-FISH showing the localization of POT1 at telomeres in HeLa 1.2.11 cells transfected with SerRS or empty vector. Cells were hybridized with Cy3-labeled (CCCTAA)_3_ PNA probe (red) and immunostained with POT1 (green). Scale bars represent 5 μm. **e** Quantification of the data in **d** to show the average percentages of POT1-associated telomere foci per cell (means ± SEM, *n* = 50, ***P<0.001, two-tailed Student’s *t*-test). **f** Quantification of the data shown in **d** to show the percentages of cells with more than ten POT1-associated telomeric foci (mean ± SEM, *n* = 3, **P < 0.01, two-tailed Student’s *t*-test)

Next, we determined whether SerRS could influence the recruitment of POT1 to telomeres, given that SerRS can directly bind telomeric DNA repeats and can interact with POT1 via the UNE-S domain, which is not involved in DNA interaction.^28^ To address this question, we overexpressed V5-tagged SerRS in HeLa 1.2.11 cells, which had a very low level of endogenous SerRS in the nucleus. We observed an enrichment of POT1 on telomeric DNA, as detected by a ChIP assay that was compared with empty vector-transfected cells (Fig. 5a, b); however, overexpression of SerRS did not change the expression of POT1 (Fig. 5c). To confirm these findings, we also examined POT1 localization at telomeres by IF and FISH staining in HeLa 1.2.11 cells transfected with the SerRS vector or the empty vector. As shown in Fig. 5d, POT1 showed significantly increased recruitment to telomeres in SerRS-overexpressing cells. Quantitative analysis indicated that the average percentage of POT1-associated telomere foci was approximately 55.3% in SerRS-overexpressing cells, which was much more than what was observed in control cells (~30%) (Fig. 5e). Additionally, a higher number of cells showed increased POT1-associated telomeres upon SerRS overexpression (Fig. 5f). Thus, nuclear SerRS can tether more POT1 protein to telomeric DNA.

### SerRS prevents the recruitment of telomerase to telomeric DNA

**Fig. 6:**
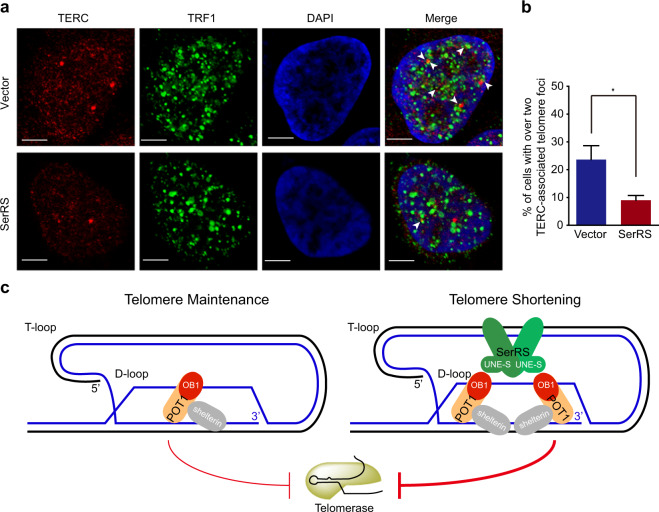
SerRS prevents the recruitment of telomerase to telomeric DNA. **a** Telomerase RNA (TERC; red) and telomeres were visualized by RNA FISH and immunofluorescent staining with anti-TRF1 antibody (green), respectively, in HeLa 1.2.11 cells transfected with the SerRS expression vector or an empty vector. Arrows indicate telomerase foci colocalizing with telomeres. Scale bars represent 5 μm. **b** Data from **a** were quantified and plotted as a bar graph (means ± SEM, *n* = 3, *P < 0.05, two-tailed Student’s *t*-test). Cells with ≥2 telomerase-associated foci were counted as positive, and more than 80 cells were examined for each cell line. **c** Model proposing a role for SerRS as a regulator of telomere length. The SerRS dimer may bind to telomeric DNA repeats, and it tethers two POT1 molecules onto a telomeric single-stranded 3’-overhang, preventing the access of telomerase and the elongation of telomere

POT1 has been reported to regulate the accessibility of telomerase to telomeric DNA through DNA binding competition.^40^ We next investigated whether POT1 tethered on telomeric DNA by SerRS can prevent the recruitment of telomerase. To test this hypothesis, we utilized FISH assays to detect the abundance of telomerase on telomeres via hybridizing the RNA component of telomerase, i.e., TERC,^41^ while the telomeres were counterstained by immunofluorescent staining of the Shelterin complex protein TRF1. HeLa 1.2.11 cells were transfected with the SerRS expression vector or an empty vector and then synchronized at the S phase, when telomerases are recruited to telomeres.^42^ We observed that overexpression of SerRS resulted in fewer telomerases being recruited to the telomeres (Fig. 6a, b). These data suggested that SerRS promoted the recruitment of POT1 to the telomere, blocking the access of the telomerase.

In summary, our data revealed a noncanonical role of SerRS in controlling telomere length. Once SerRS enters the nucleus, it can directly bind telomeric DNA repeats and tether more POT1 protein on telomeres, which prevents the engagement of telomerase, resulting in progressive telomere shortening and cellular senescence, thus leading to tumor suppression (Fig. 6c).

## Discussion

During evolution, AARSs gained many new domains and expanded their functions beyond their canonical functions in protein biosynthesis.^26^ These expanded functions make AARSs a good bridge between protein homeostasis (proteostasis) and other cellular and biological processes, such as cellular senescence and aging. Many recent studies revealed that some AARS members regulate longevity-related pathways. As a well-known example, LeuRS has been shown to regulate the amino acid signal in conserved mTORC1 signaling that is a well established major regulator of lifespan.^43^ Here, we revealed another mechanism through which AARSs can regulate cellular senescence through direct regulation of telomere length. We revealed that nuclear SerRS directly binds to telomeric DNA repeats and recruits POT1 protein to the telomere, which prevents the engagement of telomerase, and results in progressive telomere shortening and cellular senescence.

UNE-S is a unique domain at the carboxyl-terminus of SerRS in all vertebrates, from fish to humans.^44^ Our previous studies have revealed that the UNE-S domain harbors a robust NLS, which directs SerRS into the nucleus where it participates in vascular development.^28^ Here, we discovered a novel role for the UNE-S domain in the interaction with the Shelterin protein POT1, which is able to trigger telomere shortening and senescence. This result indicates more complicated biological roles were conferred to SerRS during evolution by the acquisition of a multifunctional domain.

As a member of the Shelterin complex, POT1 binds single-stranded telomeric DNA with high affinity and specificity.^39^ This binding is mediated by the two OB domains of POT1 at the N-terminal end of the protein.^45^ The domain mapping analysis showed that the OB1 domain of POT1 was required for SerRS to interact with POT1. Previous studies showed that overexpression of OB1-deleted POT1 (POT1-ΔOB) resulted in rapid telomere elongation, highlighting the important role of OB1 in POT1-mediated telomere length control.^14^ According to our results, SerRS may also be involved in this process. However, it is still unknown how the SerRS-POT1 interaction cooperates with the POT1-TPP1 interaction to regulate telomere length homeostasis. It is also unknown how proteostasis stress alters SerRS nuclear import and therefore regulates telomere length.

## Materials and methods

### Cell culture

HeLa 1.2.11, HeLa VST and 293T cells were cultured in Dulbecco’s modified Eagle’s medium (DMEM) with 4.5 g/L glucose (Biological Industries, Israel), supplemented with 10% fetal bovine serum (FBS) (Biological Industries), 100 U/mL penicillin and 100 mg/mL streptomycin (Gibco, Grand Island, NY, USA) at 37 °C and 5% CO_2_. BJ cells were kindly provided by Peiqing Sun and cultured in EMEM supplemented with 10% FBS, 100 U/mL penicillin and 100 mg/mL streptomycin.

### Tumor xenograft and immunohistochemistry

HeLa cells (6×10^6^ cells) stably expressing SerRS or stably transfected with an empty vector were injected subcutaneously into six-week-old female NOD/SCID mice to establish a mouse cervical cancer xenograft model. Tumor volumes were determined by measuring the length and width of the xenografts with a Vernier caliper and calculated using the formula (length×width^2^)/2. When mice were sacrificed, tumor xenografts from each mouse were dissected and used for further analyses. The sections of xenografts were stained with an anti-P21 antibody (Proteintech, China) at a 1:50 dilution, an anti-P16 antibody (Proteintech, China) at a 1:50 dilution and an anti-β-gal antibody (Proteintech, China) at a 1:50 dilution. After washing the samples, they were incubated with biotin-conjugated secondary antibodies, which was followed by incubation with streptavidin-HRP; finally, they were visualized with a 3,3’-diaminobenzidine (DAB) substrate (ZSGB BIO, Beijing, China). Images were taken using an Olympus microscope (Olympus, Tokyo, Japan). The H-score was used to quantify P21, P16 and β-gal expression in tumor tissues, which was calculated by multiplying the staining area (scored as 1, 2, 3 and 4; 1 for 0–25%, 2 for 25%–50%, 3 for 50%–75%, and 4 for 75%–100% positively stained areas) with the staining intensity (negative, weak, moderate and strong were scored as 1, 2, 3, and 4 based on color density, respectively). Student’s *t*-tests were performed for statistical analysis.

### Plasmids

For the construction of the pFLAG-SerRS, pFLAG-TRF1 and pcDNA6c-POT1 plasmids, human SerRS, TRF1 and POT1 genes were cloned from HEK293T cells by RT-PCR and inserted into the pFLAG-CMV2 vector (Sigma-Aldrich, St Louis, MO, USA) or the pCDNA6c vector (Thermo-Fisher Scientific, Waltham, MA, USA). For purifying recombinant SerRS and POT1, the coding sequences for SerRS and POT1 were subcloned into both the pET20b vector (Merck, Temecula, CA, USA) and the pGEX-6p-1 vector (GE Healthcare Bio-Sciences, Uppsala, Sweden).

### Analysis of senescence-associated beta-galactosidase (SA-β-gal) activity

The activity of SA-β-gal in SerRS-overexpressing BJ cells was determined using 5-bromo-4-chloro-3-indolyl P3-D-galactoside (X-gal) following the protocol described in a kit (Beyotime, China). SA-β-gal-positive cells were counted under a microscope and expressed as the % of total cells.

### Nuclear fractionation analysis

Nuclear fractionation was carried out as previously described by Xu et al.^28^ Cells were harvested after 24 h, and the cytoplasmic and nuclear fractions were separated and extracted by using a NE-PER Nuclear and Cytoplasmic Extraction Kit (Thermo-Fisher Scientific). SerRS was detected with an anti-SerRS antibody (made in our lab). An anti-Lamin A/C antibody (Cell Signaling Technology, Danvers, MA, USA) and an α-tubulin antibody (Proteintech, China) were used to identify nuclear and cytoplasmic markers, respectively.

### Telomere restriction fragment analysis by Southern blot

The telomere restriction fragment (TRF) analysis was performed using a TeloTAGGG Telomere Length Assay kit (Roche Life Science, Switzerland) with slight modifications. Briefly, 2 μg of genomic DNA was extracted from HeLa cells using a DNeasy Blood & Tissue Kit (QIAGEN, Germany) and digested with a Hinf I and Rsa I enzyme mixture overnight at 37 °C. Then, each digested DNA sample was loaded onto a 0.8% agarose gel and run at 100 V for 3 h. DNA was denatured, neutralized, and then blotted onto the nylon membrane (Sigma) by capillary transfer using 20× saline sodium citrate (SSC) buffer for 20 h at room temperature. After transfer, DNA was fixed on the wet blotting membrane by 120 mJ UV-crosslinking and hybridized with a DIG-labeled (CCCTAA)_4_ oligo probe in hybridization buffer overnight at 37 °C. Then, the membrane was washed and incubated with anti-DIG-AP (Roche, at 1:4000 dilution) and visualized with an imaging system, Tanon-5200 (Tanon, Beijin, China). The mean TRF length can be obtained by comparing the mean sizes of the smeared bands to the molecular weight markers using the GelPro analyzer.

### Coimmunoprecipitation

HeLa cells were plated in a 10-cm dish and transiently transfected with 3 μg of pFLAG-plasmid and 3 μg pcDNA6C-plasmid by Lipofectamine 2000 (Invitrogen, Carlsbad, CA, USA). After 24 h of posttransfection, cells were lysed by sonication in IP buffer (20 mM Tris–HCl pH 7.5, 150 mM NaCl, 2 mM EDTA, 0.1% Triton X-100 and protease inhibitor cocktail (Roche)). Immunoprecipitation was carried out by incubating the supernatants with an anti-FLAG antibody (Sigma, #F1804, at 1:3000 dilution) or an anti-V5 antibody (Invitrogen, #46-1157, at 1:3000 dilution), followed by incubation with protein G agarose (Thermo-Fisher Scientific) at 4 °C overnight. After being washed three times, the recombinant proteins were eluted and detected by western blotting with an anti-FLAG antibody or an anti-V5 antibody. For the co-IP of endogenous POT1 and SerRS, 2 µg of anti-POT1 antibody (Abcam, #ab194480) and 2 µg of anti-SerRS antibody were used.

### Protein expression and purification

PET-20b-SerRS and pET-20b-POT1 plasmids were used to transform *E. coli* BL21 (DE3) cells (CWBIO, Beijing, China). The expression of recombinant proteins was induced by 1 mM IPTG (Thermo-Fisher Scientific) for 8 h at 25 °C. Cells were harvested and lysed by sonication in lysis buffer (50 mM NaH_2_PO_4_ pH 8.0, 300 mM NaCl, 10 mM imidazole, 2 mg/ml lysozyme, protease inhibitor cocktail). The supernatants were incubated with Ni-NTA agarose beads (Qiagen) overnight at 4 °C. The beads were consecutively washed with wash buffer containing 20 mM and 40 mM imidazole. The recombinant proteins were eventually eluted with elution buffer containing 100 mM imidazole and were dialyzed to remove imidazole.

For the expression of GST-tagged proteins, pGEX6P-1-SerRS and pGEX6P-1-POT1 vectors were used to transform *E. coli* BL21 (DE3) cells. GST-tagged protein purification was carried out using glutathione agarose (Thermo-Fisher Scientific), and eluting was performed with phosphate-buffered saline (PBS) buffer containing 20 mM reduced glutathione (Sigma-Aldrich). The purities of the recombinant proteins were assessed by SDS-PAGE electrophoresis and Coomassie blue staining. The protein concentrations were measured by NanoPhotometer (IMPLEN, Munich, Germany).

### GST pulldown

GST pulldown assays were carried out, as previously described.^29^ Briefly, GST-fusion proteins bound to beads were incubated with 0.5 μM purified recombinant proteins in 500 µl of GST pulldown buffer (150 mM NaCl, 20 mM HEPES pH 7.9, 0.5 mM EDTA, 0.1% Triton X-100, 10% glycerol, 1 mM DTT) overnight at 4 °C. After being washed 5 times with pulldown buffer, the proteins bound to the beads were analyzed by western blot, using an anti-His (Proteintech, #66005-1-Ig, at 1:1000 dilution) or an anti-GST (Proteintech, #10000-0-AP, at 1:1000 dilution) antibody.

### Immunofluorescence (IF) and fluorescent *in situ* hybridization (FISH)

Cells grown on coverslips were fixed with 4% paraformaldehyde and permeabilized in 0.25% Triton X-100 for 10 min at room temperature. After incubation with blocking buffer (5% goat serum, 0.1% Tween-20, PBS) for 1 h, cells were incubated at 4 °C overnight with anti-SerRS (made in the lab; 1:100 dilution) or anti-POT1 (Santa Cruz, CA, USA; 1:100 dilution) antibodies. Coverslips with cells were washed three times before a 1 h incubation at room temperature with secondary antibodies (goat anti-rabbit IgG or goat anti-mouse IgG antibodies conjugated with either Alexa488 or Alexa594). Cells were then washed and stained with 0.1 μg/mL 4,6-diamidino-2-phenylindole (DAPI, Sigma-Aldrich) and mounted onto glass slides with ProlongGold Antifade Reagent (Thermo-Fisher).

For IF staining combined with telomere FISH, after fixation, permeabilization and washing with PBS, cells were dehydrated sequentially in 70%, 90%, and 100% ethanol for 2 min each. A Cy3-OO-(CCCTAA)_3_ PNA telomere probe (Panagene, Korea) was added to the glass slides in hybridization buffer (70% formamide, 10 mM Tris-HCl pH 7.2, 1×blocking reagent (Roche)). After denaturation for 3 min at 80 °C, slides were hybridized for 2 h at room temperature in a humidified chamber and then washed twice with wash buffer (70% formamide, 0.1% bovine serum albumin, 10 mM Tris–HCl pH 7.2) for 15 min each and three times with PBST (0.1% Tween 20 in PBS) for 5 min each prior to mounting and imaging.

For IF staining combined with telomerase RNA FISH, cells were fixed in 4% formaldehyde, processed in sequential dehydration solutions containing 70, 90%, and 100% ethanol, and then rehydrated in 2×SSC buffer with 50% formamide. Prehybridization was performed in a 2×SSC solution containing 10% dextran sulfate, 2 mM vanadyl ribonucleotide complex, 0.002 mg/mL nuclease-free BSA, 1 mg/mL *E. coli* tRNA, 1 U/μL RNasin, and 50% deionized formamide for 1 h at 37 °C in a humidified chamber. For *in situ* hybridization, the cells were hybridized in the prehybridization solution by incubation with a mixture of three Cy3-conjugated hTERC probes (50 ng/probe/sample) (Sangon Biotech, Shanghai, China) overnight at 37 °C. After washing sequentially with 50% formamide in 2×SSC solution at 37 °C and PBS at room temperature, the cells were incubated with a TRF1 antibody (Abcam, #ab10570, 1:100) following the IF protocols described above.

Cell images were acquired with an Olympus FV1000 microscope (Tokyo, Japan) using a 100× objective. All image files were randomly assigned coded names to allow blinded scoring for spot colocalization and fluorescence intensity quantification. The sequences for the hTERC probes are as follows: hTERC1 (5′ GCT*GACATTTTTGTTTGCTCAGAATGAACGGGGAAGGCGGCAGGCCGAG GCT*T 3′), hTERC2 (5′ CT*CCGTTCCTCTTCCGCGGCCTGAAAGGCCGAACC TCGCCCCGCCCCCGAGT*G 3′), hTERC3 (5′ AT*GTGTGAGCCGAGCCTGGG TGCACGCCCACAGCTCAGGGAACGCGCCGCGCT*C 3′). The * indicates a Cy3-conjugated T.

### Metaphase-FISH

After the cell density reached 70–80% confluence, colcemid (0.1 μg/ml, Sigma-Aldrich) was added to the medium for 2 h. Cells were harvested with trypsin-EDTA, washed with PBS, and resuspended in 75 mM KCl at 37 °C for 30 min. Then, the cells were fixed with 2 ml of fresh cold fixative solution (methanol:acetic acid, 3:1) and mixed carefully by inverting the tube. Cells were harvested by centrifugation, and the fixation step was repeated. Finally, the cell suspension was dropped onto glass slides and incubated at 80 °C for 3 min; then, the glass slides were left to dry overnight. Slides were immersed in PBS for 5 min, fixed with 2% formaldehyde and dehydrated sequentially in 70, 90, and 100% ethanol for 2 min each. A Cy3-OO-(CCCTAA)_3_ PNA telomere probe or a FITC-OO-(CCCTAA)_3_ PNA telomere probe was added to the glass slides in hybridization buffer containing 70% formamide, 10 mM Tris­HCl pH 7.2, 1×blocking reagent and PBS. After denaturation for 3 min at 80 °C, slides were hybridized for 2 h at room temperature in a humidified chamber, washed twice with a solution of 70% formamide, 0.1% bovine serum albumin, and 10 mM Tris-HCl at pH 7.2 for 15 min each, and then washed three times with TBST for 5 min each. Glass slides were dehydrated sequentially with 70, 90, and 100% ethanol again, counterstained with 0.1 μg/mL DAPI, and then mounted onto glass slides with ProlongGold Antifade Reagent. Photos were taken using an Olympus FV1000 fluorescence microscope. The number of signal-free ends per cell in metaphase was determined by manual inspection of the same metaphase images that were used for telomere signal-intensity quantification. Statistical significance was analyzed with a two-tailed Student’s *t* test.

### Q-FISH

Metaphase-FISH was performed as described above. Quantitative-FISH analysis was performed using TFL-TELO image analysis software. The distributions of fluorescence intensities, which are shown in arbitrary fluorescence units, of individual telomeres from a total of 20 cells in metaphase per treatment were displayed.

### Chromatin immunoprecipitation (ChIP) and Dot-blot

ChIP was carried out using a ChIP-IT Express Enzymatic Kit (Active Motif). Briefly, cells grown to 70–80% confluence were cross-linked with 1% formaldehyde for 10 min; crosslinking was stopped by the addition of glycine to a final concentration of 0.125 M, which was followed by incubation for 5 min. Pellets were collected by scrapping cells and centrifuging them for 10 min at 2500 r/min and 4 °C. After cell pellets were lysed, the nuclei pellets were resuspended in digestion buffer and incubated with 17 μl of 1× enzymatic shearing cocktail at 37 °C for 5 min. The sheared chromatin was incubated overnight at 4 °C with 35 µl of protein G magnetic beads and 2 μg of anti-Flag antibody (Sigma-Aldrich, #F1804) or mouse IgG (Abmart, #B30010M) as a negative control. After washing, immunoprecipitated chromatin was eluted with elution buffer for 15 min and reverse-crosslinked by treatment with 5 M NaCl at 95 °C for 15 min. DNA was purified with RNase A and proteinase K treatment for 1 h at 37 °C, denatured at 95 °C for 5 min and immediately placed on ice. The DNA samples were blotted using a Bio-Dot Microfiltration Apparatus (GE Healthcare, Little Chalfont, UK) and hybridized with a digoxigenin (DIG)-labeled probe (Sangon Biotech, Shanghai, China) that is specific for telomeric repeats. The signal intensity was measured by GelPro software and was normalized to the signal from the input DNA on the same blot.

### Electrophoretic mobility shift assay (EMSA)

EMSA was performed as Xu et al. described.^29^ Briefly, telomeric [TTAGGG]_5_ dsDNA were synthesized, annealed, and [^32^P]-labeled at the 5′ end by T4 DNA kinase (New England Biolabs) before purification using a sephadex G-25 spin column (GE Healthcare). The labeled oligonucleotides (0.8 pmol) were incubated with recombinant SerRS at the indicated concentrations in binding buffer (20 mM Tris-HCl pH 8.0, 60 mM KCl, 5 mM MgCl_2_, 0.1 mg/mL BSA, 10 ng/µl poly (dG-dC), 1 mM DTT) for 30 min at room temperature. The samples were loaded onto a 5% native polyacrylamide gel and underwent electrophoresis at 250 V in running buffer (25 mM Tris pH 8.3, 190 mM glycine). Then, the gel was dried and examined by autoradiography.

Oligonucleotides used for EMSA are as follows: Telomere: 5′ TTAGGGTTAGGGTTAGGGTTAGGGTTAGGG 3′ (top strand) and 5′ CCCTAACCCTAACCCTAACCCTAACCCTAA 3′ (bottom strand); Control: 5′ TCGAAGTCGAAGCATGGGTCGAAGCATGGG 3′ (top strand) and 5′ CCCATGCTTCGACCCATGCTTCGACTTCGA 3′ (bottom strand); *VEGFA* promoter: 5′ GGCGGGGCGGAGCCATGCGCCCCCCCCTTT 3′ (top strand) and 5′ AAAGGGGGGGGCGCATGGCTCCGCCCCGCC 3′ (bottom strand).

### Western blot analysis

Cells were lysed in cold lysis buffer supplemented with protease inhibitor cocktail, and the lysate was subjected to SDS-PAGE. Proteins were transferred onto nitrocellulose membranes (Millipore, Billerica, MA, USA) and blotted with antibodies as follows: anti-SerRS (made in lab, 1:2000), anti-POT1 (Abcam, 1:500), anti-GST (Proteintech, 1:1000), anti-Flag (Sigma, 1:3000), anti-V5 (Invitrogen, 1:3000), anti-β-actin (Santa Cruz, 1:5000), anti-P21 (Santa Cruz, 1:1000), anti-P16 (Abcam, 1:1000), and anti-β-gal (Proteintech, 1:1000). Proteins were visualized by enhanced chemiluminescent (ECL) reagent (Thermo-Fisher Scientific).

## Supplementary information

Supplementary Material
