# Supplementary material for: Seryl tRNA synthetase cooperates with POT1 to regulate telomere length and cellular senescence
Source: Signal Transduct Target Ther. 2019 Nov 29;4:50. doi: 10.1038/s41392-019-0078-1 (PMC6882858; doi:10.1038/s41392-019-0078-1)
Supplement: Supplementary file 1 — Supplementary Material [file 41392_2019_78_MOESM1_ESM.docx]

**Supplementary Materials for**

**Seryl tRNA synthetase cooperates with POT1 to regulate the length of telomere and cellular senescence**

Yingxi Li^1^, Xiyang Li^1^, Mei Cao^1^, Yuke Jiang^1^, Jie Yan^1^, Ze Liu^1^, Rongcun Yang^1^, Xu Chen^2^, Peiqing Sun^3^, Rong Xiang^1^, Longlong Wang^1, 2*^and Yi Shi^1, 2*^

^1^School of Medicine, Nankai University, 94 Weijin Road, Tianjin 300071, China

^2^Tianjin Key Laboratory Human Development and Reproductive Regulation, Nankai University Affiliated Hospital of Obstetrics and Gynecology, Tianjin, China

^3^Department of Cancer Biology, Wake Forest Comprehensive Cancer Center, Wake Forest School of Medicine, Winston-Salem, NC, USA

*Correspondence to: Yi Shi ([yishi@nankai.edu.cn](mailto:yishi@nankai.edu.cn)) and Longlong Wang ([wangl@nankai.edu.cn](mailto:wangl@nankai.edu.cn)), Phone: (86)-22-23509482, Fax: (86)-22-23502554

**Running title**: SerRS binds POT1 to control telomere length

Conflicts of interests: The authors declare no competing interests.

**This PDF file includes:**

Figures. S1

**Figure S1**

**
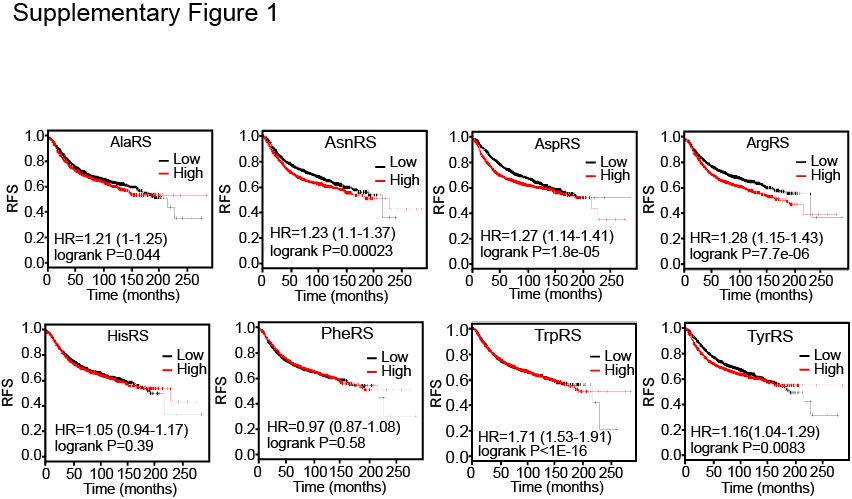
**

**Figure S1.** Kaplan-meier plots and hazard ratio analysis of human tRNA synthetases without tight correlation with the relapse-free survival (RFS) of breast cancer patients. Patient samples were divided in two halves as “low-expression (black)” and “high-expression (red)” sets for each tRNA synthetase in the analysis (n=1764).
